# Combination of mechanical debridement and interleukin 12 or interleukin 23 therapy for refractory facial pyoderma gangrenosum with NOD2 mutation

**DOI:** 10.1016/j.jdcr.2025.06.019

**Published:** 2025-06-23

**Authors:** Morgan Vague, Moira Shea, Shivani Thacker, Heather Onoday, Alex G. Ortega Loayza

**Affiliations:** Department of Dermatology, Oregon Health & Science University, Portland, Oregon

**Keywords:** immunodermatology, inflammation/inflammatory, lasers, pyoderma gangrenosum, scars, wounds and wound healing

## Introduction

Pyoderma gangrenosum (PG) is a rare neutrophilic dermatosis that manifests as painful, nonhealing ulcers. The rarity of PG, combined with its variable clinical presentation, makes timely diagnosis challenging, and the lack of Food and Drug Administration approved therapies limits treatment options. The Th1/Th17, JAK/STAT, TNF-α, and interleukin (IL)12 or IL-23 pathways are implicated in PG pathogenesis and associated autoinflammatory comorbidities such as inflammatory bowel disease (IBD) and rheumatoid arthritis and offer valuable insights into targeted PG treatment through off-label biologic therapy.^1^, ^2^, ^3^ Patient response to biological therapy remains variable, however, highlighting the crucial utility of genetic testing in both identifying genetic aspects of PG pathogenesis and guiding targeted PG treatment.

## Case report

**Fig 1:**
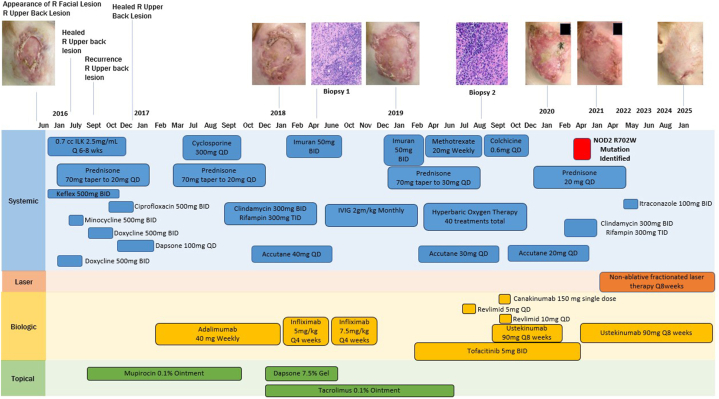
A timeline of the patient’s treatment history and target ulcer healing. Biopsy 1 and 2 consisted of 3 mm punches from perilesional skin. Histology images from biopsy 1 show mixed histiocyte, lymphocyte, and neutrophil infiltration. Histology from biopsy 2 show superficial mixed neutrophilic infiltrate. (Original magnifications: biopsy 1, ′5; biopsy 2, ′20)

Here we present the case of a 23-year-old individual diagnosed with PG of the right face at the age of 15 years with no personal or familial history of underlying autoimmune conditions. The patient was referred following unsuccessful surgical excision of 2 golf-ball-sized nodules, initially diagnosed as infundibular cysts, that grew into nonhealing lesions on the right face and right upper back. Infectious disease workup resulted in multiple antibiotic treatments (Fig 1) in the setting of negative bacterial cultures, but the affected areas remained unhealed. Subsequently, the patient’s right upper back ulcer healed following 3 injections of intralesional triamcinolone 10 mg/mL, but the facial ulcer persisted.

PG was diagnosed using the validated PARACELSUS criteria. There were tunneling nodules, neutrophilic infiltrate, and the presence of sinus tract formation on perilesional punch biopsy of the right cheek (Fig 1). Differential diagnoses included cutaneous manifestations of IBD and granulomatosis with polyangiitis; however, colonoscopy and anti-neutrophil cytoplasmic antibody testing were negative. The patient underwent 14 different systemic therapy courses over a period of 6 years without success (Fig 1). This prompted genetic testing via the Invitae Primary Immunodeficiency Panel that revealed a heterozygous NOD2 R702W mutation. A second perilesional biopsy of the right face showed a neutrophilic infiltrate and a histiocyte-rich infiltrate indicative of granulomatous inflammation.

The NOD2 R702W mutation implicated the IL-12/IL-23 pathway as a salient treatment target. Although no Food and Drug Administration-approved treatments exist for PG, the IL-12/IL-23 inhibitor ustekinumab is Food and Drug Administration-approved for the treatment of IBD. The patient had previously undergone an unsuccessful combination therapy of ustekinumab 90 mg subcutaneous loaded at week 0 and week 4, followed by every 8 weeks in conjunction with Janus kinase inhibitor tofacitinib 5 mg BID for 6 months; however, superficial wound culture of the affected area was positive for mucormycosis, suggesting excessive immunosuppression (Fig 1). Identification of a NOD2 R702W variant guided the reinitiation of ustekinumab monotherapy at 90 mg subcutaneous loaded at weeks 0 and 4, followed by every 8 weeks. Ustekinumab monotherapy led to control of the patient’s systemic inflammation and sustained healing of the target ulcer for 3 years (Fig 1). Systemic inflammation control enabled the use of ablative mechanical and nonablative laser therapies to address aspects of facial scarring once ulceration was no longer present. Upon healing, the patient presented with an 8 × 5 cm erythematous, cribriform scar with undermined borders and irregular papules, and irregularly distributed ridged contours. Low-level (1) electrocautery was first used to address the irregular topography of the scar through papule ablation. This was combined with tangential excisional scissor removal of the irregular ridged contours, resulting from healed, scarred tissues. Nonablative fractionated laser therapy was initiated at 1550 nm with a fluence of 45 J/cm^2^ for 8 passes; treatment level (7) every 8 weeks to remodel the collagen of the wound bed, leading to a significant improvement in scarring following healing (Fig 1).

## Discussion

This case highlights the role of genetic testing to guide targeted biologic treatment of PG, particularly at an early disease stage. Despite the patient’s negative colonoscopy, the well-documented association of IBD with the patient’s identified NOD2 R702W variant guided the successful reinitiation of ustekinumab monotherapy.^4^ Targeting the IL-12/IL-23 pathway enabled effective control of the patient’s systemic PG inflammation.^5^ Additionally, the granulomatous inflammation present in the patient’s second perilesional biopsy is associated with NOD2 dysregulation, which has been increasingly implicated in autoinflammatory conditions.^6^ Recent studies have implicated mutations in the NOD2 gene as inborn errors of immunity associated with PG pathogenesis and atypical PG presentation.^7^ In a genetic profiling study of lesional skin from PG patients, 9 of 13 individuals harbored NOD2, including 1 case with the NOD2 R702W variant.^8^ Taken together, this highlights a potential role for NOD2 dysregulation in PG pathogenesis and underscores the utility of genetic testing in identifying disease mechanisms and optimizing therapies for PG.

In cosmetically sensitive areas, mechanical therapies can significantly optimize patient outcomes. Control of the active inflammatory phase of PG is necessary to transition to the healing phase, and sustained PG healing is crucial to a setting amenable to mechanical scar reduction therapies.^9^ Although current research into mechanical therapy and scar reduction in PG is limited to short-term improvement,^10^ this case demonstrates the use of genetic testing to guide sustained PG healing and create the necessary conditions for long-term improvement in PG scar appearance using mechanical therapy. The patient’s long-term improvement highlights the broader implications of genetic testing in PG, not only for targeting systemic therapy but also for expanding treatment possibilities, including mechanical interventions, to improve scarring and patient outcomes.

## Conflict of interest

Ms Vague, Mrs Shea, and Drs Thacker, and Onoday have no conflict of interest to report. Dr Loayza is the former President of the Pacific Dermatology Association, serves as an associate editor for *Dermatology (Karger)*, and is an editorial board member of the *American Journal of Clinical Dermatology*. He is also a consultant for Genentec, Guidepoint, Inflarx, Corvus Pharmaceuticals, Castle Biosciences, Clarivate, TFS Healthscience, Otsuka, and Leo Pharma. He is an advisor to Bristol Meyer Squibb, Boehringer Ingelheim, Janssen, and Sanofi. Dr Loayza has received research grants from Lilly, Janssen, and Pfizer.
